# Recognition and management of acute functional decline in older people living in care homes: a qualitative interview study with UK care home staff

**DOI:** 10.3399/BJGPO.2024.0252

**Published:** 2025-09-24

**Authors:** Abigail Moore, Margaret Glogowska, Christopher Butler, Gail Hayward

**Affiliations:** 1 Nuffield Department of Primary Care Health Sciences, University of Oxford, Oxford, UK

**Keywords:** qualitative research, nursing homes, aged, urinary tract infections

## Abstract

**Background:**

Older people living in care homes who experience acute functional decline pose a diagnostic challenge to GPs.

**Aim:**

We aimed to explore beliefs, practices, and experiences of UK care home staff who first recognise and respond to acute functional decline, including in the context of the COVID-19 pandemic.

**Design & setting:**

Qualitative interview study with 25 UK care home staff.

**Method:**

Semi-structured interviews were conducted by telephone between January 2021 and April 2022. Thematic analysis was facilitated by NVivo software.

**Results:**

Care home staff recognised acute functional decline as subtle changes from normal, which required knowing a resident well. However, it could be difficult to differentiate between an *‘off day’* and a more significant deviation, particularly for residents with a variable baseline. Acute functional decline caused anxiety to care home staff, in part due to the uncertainty about illness trajectory and outcome. They commonly considered urinary tract infection (UTI) to be a likely underlying cause. Some participants described a watch-and-wait approach or trying simple interventions, while others preferred escalating directly to outside clinical support. Triggers for escalation included perceived severity of illness, gut feeling, or failure to respond to initial supportive management.

**Conclusion:**

This study has highlighted the complexities around the identification and management of a care home resident experiencing acute functional decline. There was variation in interpretation and responding to these episodes within the care home. More work is needed to understand the physiology and risk profiles of acute functional decline, as well as any relationship to UTI.

## How this fits in

Older people living with frailty, including those residing in care homes, can experience a functional decline when experiencing acute illness. Little is known about how care home staff first recognise and respond to these episodes. Our study has explored care home staff beliefs about underlying causes of acute functional decline, which they commonly associate with urinary tract infection (UTI). It is useful for GPs to recognise that significant work may have been done by care home staff in monitoring and managing the resident before they seek outside advice.

## Introduction

Approximately 380 000 older people live in care homes in the UK^1^ and many live with frailty, multimorbidity, and cognitive impairment.^2^ They require greater input from GPs^3–5^ and are more likely to be admitted to hospital compared to older people living in the community.^6–8^ In addition, care home residents are more frequently prescribed antibiotics^9^ and are colonised with a higher proportion of antibiotic-resistant bacteria compared to their community-dwelling counterparts.^10^

Older people living with frailty, including those residing in care homes, can experience a functional decline when experiencing acute illness.^11^ This can include physical symptoms like fatigue, loss of appetite, changes in mobility or activities of daily living, and/or changes in cognition.^12^ We know that GPs face diagnostic uncertainty^13^ when managing these non-specific presentations, which may then result in antibiotic prescribing,^14^ even though infection is just one of many possible causes.^15–17^

However, upstream to healthcare professionals are the care home staff initially recognising and responding to acute functional decline and little is currently known about their understanding of these episodes. In this qualitative interview study, therefore, we aimed to explore beliefs, practices, and experiences of the care home staff recognising and responding to acute functional decline in the older people they care for, including in the context of the COVID-19 pandemic.

## Method

We used the principles of grounded theory as a guide to our data collection and analysis.^18^

### Recruitment

Staff aged ≥18 years, currently working in a care home for older adults in England, Scotland, or Wales for at least a year, were recruited using advertisements circulated via a mixture of social media, research mailing lists, direct contact with care homes, and snowballing. Care home staff were ineligible if they could not participate in an interview in English.

Sampling was purposive, targeting care home staff with a range of roles, experience, and geographical location to achieve maximum variation. Participants were offered an interview at a time convenient to them and were reimbursed with a gift card for their participation.

Recruitment continued until the study team agreed that there was sufficient information power. This was when no amendments to the topic guide were considered necessary, when no new codes or significant themes were emerging, and the team felt that there was sufficient detail to explain the themes.^19^

### Data collection

AM, a female academic GP with an interest in care homes and trained in qualitative research methods, conducted the interviews by telephone between January 2021 and April 2022. None of the participants were known to AM beforehand. Participants were told the aims of the study and gave verbal, recorded informed consent.

Interviews were semi-structured, following a flexible topic guide developed and pilot tested by the research team. Interviews lasted between 60 and 80 minutes, were audio-recorded, and transcribed verbatim by a professional transcriber.

### Data analysis

Coding and analyses of the data were completed by AM using NVivo (version Mac Release 1) software to support data management. The interview transcripts were open coded and related codes were combined to create categories. Material within the categories was subsequently reviewed for explanation, before looking at how the categories combined to support emerging narratives. Earlier interviews were re-coded in the light of ongoing analysis and early analysis brought out topics that were added to the topic guide.

AM established an audit trail from the raw data of the interview transcripts through coding to development of themes to ensure dependability. The research team discussed the initial coding framework, as well as ideas for categories emerging from the data, and, subsequently, the final themes to ensure their credibility and confirmability. Group discussions with AM allowed reflexive practice throughout the process.

## Results

### Sample

In total, 47 care home staff responded to the advertisement. Of these, three did not meet the inclusion criteria for the study and 19 did not respond after receiving further information. The remaining 25 care home staff took part in interviews. (Table 1)

**Table 1.** Participant characteristics

Characteristic			*n*
Gender	Female		23
	Male		2
Job Role	Without nursing qualification	Carer	3
		Senior Carer	3
		Manager	7
		Other	1
	With nursing qualification	Nurse	2
		Advanced nurse practitioner	1
		Deputy manager	2
		Manager	6
Years’ experience in care homes	1-5		5
	6-10		5
	11-15		3
	16-20		5
	21-25		1
	>25		5
Type of care home	Residential		8
	Nursing		9
	Combined residential and nursing		7

### Findings

Themes emerging from interviews with participants are summarised in Table 2 and are explored in more detail below.

**Table 2.** Themes and subthemes

Theme	Subtheme
Recognising residents with acute functional decline within the care home	Noticing changes from normal
	Describing and communicating about the episode
	Feeling anxious about the resident
Early response to residents with acute functional decline within the care home	Sharing or shifting responsibility
	Assessing the resident
Monitoring residents with acute functional decline within the care home	Observing residents
	Triggers for seeking additional clinical support
	Contacting GPs or other sources of clinical support

### Recognising residents with acute functional decline within the care home

#### Noticing changes from normal

All interviews opened with an opportunity for participants to describe what they understood by *‘acute functional decline’*. In response, participants tended to list a series of changes in both behaviour and activities of daily living. Some participants also mixed in specific symptoms or clinical signs of acute illness, mainly those associated with infection:

*‘Mobility going down, off their food, nausea, pallor, breathlessness, if they complain of pain or discomfort, going to the toilet. If they are actually hot to touch … ’* (Participant [P]10*,* female, manager, nursing background, combined nursing/residential home, >25 years’ experience)

Participants said that sometimes the changes they noticed were subtle and required knowing the resident well. Several highlighted that some residents exhibited symptoms unique to them; examples included a flushed face or a painful neck. Most participants felt confident that they could detect an acute functional decline, with some attributing this to their years of experience of working in care homes, and some to their clinical training. However, many participants said it was the carers with no clinical training that were best placed to notice a change because they interacted the most with residents:

*‘Because you’re there a lot with those people, you get to know their likes, their dislikes, their routines and if they veer from any of that you know there’s something wrong.’* (P1, female, manager, nursing background, residential home, >25 years’ experience)

One challenge identified by some participants was recognising a change in residents who normally fluctuated day to day, like some people living with severe dementia. In these residents, it was difficult to differentiate an acute functional decline from a ‘*bad day’* (P2). Several participants characterised bad days or *‘off days’* (P15) as short-lived and less severe episodes, with residents returning to baseline quickly.

#### Describing and communicating about the episode

All participants were asked about the terms that they would use to describe or communicate to each other about residents who were experiencing an acute functional decline. Many participants gave examples of phrases that indicated that a resident was different to normal, like *‘not themselves’* (P4), *‘a bit off’* (P13)*,* or *‘they’re just not right’* (P20). Others used words that indicated ill health like *‘poorly’* (P14) or *‘generally unwell’* (P19). Some participants just talked about having and sharing *‘concerns’* (P8).

Several participants reflected how the language used within the care home differed to that used when communicating with healthcare professionals:

*‘So, the carer would report it as, as that, "oh I’m a bit concerned about the resident today," and then obviously describe their concerns. When we are talking to the doctors, to medical professionals we probably would then say there’s been an acute decline.’* (P8, female, manager, non-nursing background, residential home, 20–25 years’ experience)

A few participants explored the potential mismatch between the language used within the care home and the potential severity of the decline or the illness the resident was experiencing. This could be a barrier when communicating to healthcare professionals:

*‘It can be a bit frustrating because it’s like "well, they’re just saying they don’t feel right" they said but and then you’re thinking "well I can’t kind of present this to a GP as if they don’t feel right when there’s nothing clinically telling me that there isn’t anything wrong."’* (P15, female, deputy manager, nursing background, combined nursing/residential home, >25 years’ experience)

As a result of this mismatch, one participant had introduced training to improve interprofessional communication. In contrast, another participant felt that it was important to retain the use of simple language within the care home because most of the staff did not have a background of clinical training:

*‘We don’t like to use too much medical jargon and work just work around them. It can, it can instil a bit of fear sometimes, I think. I think it’s important that they understand as much as you because I am the only medical trained person in the home … ’* (P24, female, advanced nurse practitioner, combined residential/nursing home, 11–15 years’ experience)

#### Feeling anxious about the resident

Several participants talked about their anxiety for residents when they were experiencing an acute functional decline. For some this was because episodes did not all follow the same trajectory or have a predictable outcome. Participants talked about how sometimes an acute functional decline turned out to be just an *‘off day’*, in others it was an early sign of an acute but potentially reversible illness, while in others, it could mean that the resident was approaching end of life.

For some participants the anxiety was also driven by a close relationship with the affected resident. Others talked about the ongoing emotional impact from their experience in the early stages of the COVID-19 pandemic. The uncertainty and fear for residents appeared to impact some participants’ responses to episodes, including having a low threshold to escalate:

*‘We get quite worried when our residents get like UTIs and stuff because obviously if they’re left untreated, if any infection is left untreated it could turn sepsis and, obviously, we don’t want that … ’* (P4, female, senior carer, residential home, 6–10 years’ experience)

### Early response to residents with acute functional decline within the care home

#### Sharing or shifting responsibility

Many participants talked about sharing what they had observed about a resident quickly among their team. This could either be to validate what they had noticed or to escalate to someone more senior who could support them. Some described not wanting to hold any responsibility for a potentially unwell resident in case something went wrong, while some described a culture of automatically escalating everything to someone with clinical training:

*‘So, if it was noticed by a carer, for example, it’d go to the senior. If the senior was unsure, which 90 per cent of the time, they know how to handle it, they’d come up to management, but it then be discussed and it would go to our advanced nurse practitioner.’* (P18, female, manager, non-nursing background, residential home, 6–10 years’ experience)

Escalation appeared to alleviate anxiety for participants and removed the sense of having personal responsibility. However, one participant highlighted how a low threshold for escalation within the care home had the potential to over-burden the limited nursing staff available:

*‘The care staff certainly know when to escalate. In fact, to some extent, it probably is the opposite way that care staff will escalate too much; somebody clears their throat, they’re running down to the clinic to tell the nurse.’* (P13, female, manager, nursing background, nursing home, 21–25 years’ experience)

#### Assessing the resident

Most participants described how a resident who had been identified as experiencing an acute functional decline would be assessed at an early stage. During this assessment, most participants said a set of clinical observations would be taken. Some participants went on to use these to calculate a score, using National Early Warning Score 2 (NEWS2)^20^ or the Recognise Early Soft Signs, Take Observations, Respond, Escalate (RESTORE2) tool.^21^ Participants said that observations helped them to get an initial sense of the severity of any illness and were reassured if they and/or the early warning score were normal. There were several examples of upskilling of non-clinical staff to take observations as a result of the COVID-19 pandemic.

Many participants also said that they would collect a urine sample from the resident and do a urine dipstick as part of their initial assessment. This was because a UTI was perceived by them to be a common or usual cause for an acute functional decline. Some participants said they would do a urine dipstick for everyone, while others qualified that they would only collect a urine sample if there were urinary symptoms reported. Others felt that GPs expected a urine dipstick to be done. However, there were also participants who said that they were unclear whether a urine dipstick was recommended in current guidelines:

*‘If their urine is a bit iffy, I would also probably do a urine dip, but I mean we’ve been given so much conflicting advice on whether that’s helpful or not in people over the age of 65.’* (P7, female, senior carer, residential home, 1–5 years’ experience)

Some participants with a nursing qualification talked about doing a more thorough clinical assessment. This might involve a physical examination, as well as ruling in or out what they thought were other possible causes for an acute functional decline. Participants mentioned constipation, dehydration, medications, pain, or psychological triggers. Several participants talked of the increased responsibility the care home took for looking after the resident before escalating to the GP because of the limitations on home visits during the COVID-19 pandemic.

Doing a lateral flow test for COVID-19 had become routine in the assessment of unwell residents for many participants at the time the interviews were conducted. Several participants described how during the pandemic they had worried all the time that their residents could have COVID-19 and one participant felt that their clinical judgment had been compromised by this fear. In contrast, several participants felt that the pandemic had not changed the way in which they responded to residents with an acute functional decline.

### Monitoring residents with acute functional decline within the care home

#### Observing residents

Some participants said they would adopt a *‘watch and wait’* (P7) approach, especially if presentation was perceived to be less severe (for example, reduced food intake), the clinical observations were normal, and if there were no particularly concerning features on their assessment. This generally meant a period of closer monitoring with repeated checks of clinical observations, often coupled with encouraging oral fluids. Some nursing staff described initiating other interventions during this time including giving analgesia, antipyretics, and, if constipated, laxatives:

*‘In the meantime, we would keep an eye on them. The odd just basic things like start a fluid and food chart to make sure they were hydrated and check their output and we would, we would start that as well to monitor.’* (P10, female, manager, nursing background, combined nursing/residential home, >25 years’ experience)

The purpose of this approach was, for some of these participants, to help them differentiate a potential *‘off day’* (P15) from something more serious. Allowing a short period of time to pass gave those who were going to get better anyway the opportunity to do so and/or any simple interventions a chance to work. Some participants felt it was positive that this could prevent unnecessary GP contacts.

Some participants described when they would seek advice for those who were not getting better on the ‘watch and wait’ approach. The length of time participants allowed before seeking additional clinical support in these cases ranged from a few hours, to the next shift, and in one home there was a *‘rule of thumb’* (P7) of 3 days.

#### Triggers for seeking additional clinical support

All participants described potential triggers for wanting to seek additional clinical support for the resident. Participants had different tolerance levels to exposing themselves to risk. Those without clinical training tended to want to escalate sooner:

*‘We’ve been told by our manager that if we’re not sure with something, we should report it straight away because then if something does happen, we’re, we’re covered in the fact in that we have reported it but it was their, the professional’s decision not to do anything else about it.’* (P4, female, senior carer, residential home, 6–10 years’ experience)

Some participants said that it was the perceived severity of the illness or their own anxiety that prompted escalation. For some, severity was indicated by the speed or degree of the change from normal. For others it was *‘red flag’* (P7) symptoms like total loss of mobility or extreme confusion. Participants with a nursing background described sometimes having a *‘gut feeling’* (P12) to escalate. They said that this feeling was driven by their experience of similar episodes and/or knowing the resident well:

*‘I do believe that nurses have a sixth sense where they do pick up on things and I don’t think it’s always possible to put it into words why you think something’s not right.’* (P12, female, manager, nursing background, nursing home, >25 years’ experience)

In homes that had adopted early warning score tools, like NEWS2, the overall score or changing score would determine what they did next, including escalation to a GP or ambulance service.

#### Contacting GPs or other sources of clinical support

Participants had varying experiences of contacting GPs about unwell residents. Some had a good relationship with their usual GP, while others described challenges in communication. Several participants felt the need to be *‘forceful’* (P1) in getting their point across. Others talked about the need to be succinct and how they had learned to deliver information GPs would expect:

*‘They usually would expect us when we ring, we are prepared when we ring the doctor, so we, we have a list of who needs a GP and next to them is observations and, obviously, if we need to dip urine, we dip urine prior to ringing the doctor so we have all that information, we have the facts. It just makes the whole process a lot easier.’* (P19, female, manager, non-nursing background, residential home, 16–20 years’ experience)

Several participants talked about how the early warning score tools had improved communication with GPs and given them greater confidence in explaining the potential severity of illness, as well as being a way of avoiding lay terminology:

*‘So being able to say, “Their NEWS is at 6,” that’s like such a shorthand of saying, they’re not quite right and I also semi know what I’m doing so please listen. It’s just been brilliant, it’s been a brilliant tool.’* (P7, female, senior carer, residential home, 1–5 years’ experience)

As these interviews were carried out towards the tail end of the pandemic, many assessments with GPs were carried out over the telephone with care home staff or as a video call. Some participants explained that GPs appeared to rely on their judgement when making a clinical diagnosis, for example, accepting their assessment that a person might have a UTI. Others talked about the usual response of the GP to prescribe antibiotics for residents with an acute functional decline. Several participants described frustration at the lack of face-to-face input or investigation by GPs during this period.

There were several examples of alternative care models using remote specialist nurses who were able to advise on next steps. Advice could include trying the ‘watch and wait’ approach, simple interventions, or escalation to a GP. Participants with access to these services valued the specialist support and the quicker response compared to contacting their GP directly. Some care homes had directly employed advanced nurse practitioners who were able to provide rapid ‘in-house’ assessments and, in one case, prescribe medication. In these care homes, GPs were rarely involved in the assessment and management of residents experiencing an acute functional decline.

## Discussion

### Summary

Care home staff described acute functional decline as having a diverse illness course. Some described difficulties distinguishing transient fluctuations in functional ability, which may be normal for some residents from a more significant deviation. The uncertainty around the illness trajectory caused anxiety for care home staff during these episodes, which could result in careful monitoring and/or early escalation. Clinical observations were often measured as part of an initial assessment, sometimes to calculate an early warning score. Participants described commonly checking a urine dipstick, driven both by a belief that acute functional decline is often caused by UTI as well as anticipating a request from the GP when escalated. There were some participants with nursing training who considered a broader range of differentials for acute functional decline and several examples of more advanced assessments being done by care home staff, including simple interventions. There were also cases where advanced nurse practitioners were involved in the clinical management of these residents within the care home, without necessarily involving a GP.

### Strengths and limitations

This study provided new insights from UK care home staff exploring their understanding of a salient topic in their own terms. We were successful in recruiting participants with a range of roles and experience from care homes across the UK. However, a limitation of this study was that we did not interview the GPs linked to our participants to understand both sides of the negotiation of care for residents. For example, it is unclear whether the expectation of GPs and/or their usual response in prescribing antibiotics contributed to our participants’ belief that UTI was usually the cause of an acute functional decline.

It is important that the context of the COVID-19 pandemic is considered when assessing the transferability of the results. Early interviews in the study took place during the third national lockdown in the UK, and the final interviews were carried out when the last remaining government-enforced restrictions on social distancing were being relaxed. However, as described in the results, the impact of the pandemic was explored during the interviews; some participants felt that little had changed in their recognition and assessment processes.

### Comparison with existing literature

Some of the themes in this study aligned with those identified by a recent scoping review on care home staff recognition of acute deterioration (a sudden decompensation of physiological and/or mental status that could necessitate hospital admission).^22^ This review also highlighted the importance of the knowledge of a resident’s baseline, the transfer of responsibility up the hierarchy within the care home, the recording of clinical observations, and use of early warning scores as communication tools.

The variation in practice around using tools for recognition and communication of acute deterioration in care homes in the UK has also been previously described.^23,24^ Critically high NEWS2 scores measured in care home residents on admission can predict poor outcomes,^25^ but it is unclear how well the score performs when measured in care homes, or how it can be practically adopted. For example, a recent Norwegian qualitative study with care home staff and GPs described the limitations of using early warning scores for acute functional decline in care homes, including how some of the recommendations, like repeat observation checks, were not feasible for the setting.^26^

Our participants described how the language used by staff in care homes to describe an unwell resident could result in challenges when communicating with healthcare professionals. This is something that communication tools like Situation, Background, Assessment, Recommendation, Decision (SBARD), which is embedded in the RESTORE2 tool,^21^ are meant to solve. However, a systematic review concluded that there is limited high quality evidence to show that it is effective at improving patient safety^27^ and, in a recent study, was shown not to improve communication between care home staff and clinicians in the US.^28^

Our study added further insights into the care of UK care home residents in the post-pandemic era and explored more of care home staff beliefs about underlying causes of acute functional decline, particularly around association with UTI. From our participants’ descriptions, acute functional decline is a continuum of clinical presentations ranging from subtle changes from baseline to a severe acute deterioration. Interpretation of these changes is complex and must take into account a knowledge of the resident and their usual pattern of functional changes.

### Implications for research and practice

It is useful for GPs to understand the expertise that care home staff have on their own residents and to recognise that significant work may have been done in monitoring and managing the resident before they seek outside advice. Our results also showed there is variety in care home responses to acute functional decline, which, in some cases, can be risk averse. This is perhaps unsurprising given many care home staff have no formal clinical training. It may be helpful to consider strategies to support the care home workforce to be able to retain knowledge and good practice, and to improve consistency across care homes. It may also be useful to explore whether there are measures that could reduce uncertainty and resultant anxiety in these cases. This could involve earlier access to allied healthcare professionals and/or more point-of-care diagnostic testing. There are limited studies to date involving point-of-care testing in care home settings; one Dutch study has shown that a point-of-care C-reactive protein could safely reduce antibiotic prescribing for lower respiratory tract infection.^29^

Our study indicated that care home residents with acute functional decline vary considerably in presentation, illness trajectory, and outcome, which means that it is challenging to explore from a research perspective. Further work is needed to better describe and potentially phenotype these presentations. Knowing that care home staff associate acute functional decline with UTI is an important first step towards improving differential diagnosis of these presentations and reducing unnecessary antibiotic use by healthcare professionals in any onward contacts.

This study highlighted the complexities around the identification and management of care home residents experiencing an acute functional decline from the perspective of care home staff. More work is needed to better understand the physiology of these episodes in the care home setting, how to delineate the potential different subtypes and risk profiles (for example, subtle functional changes versus physiological deterioration), and its relationship to UTI. Understanding more about the different causes and outcomes of these episodes could empower care home staff, reduce anxiety, and may help direct diagnostic testing and/or interventions that could result in better outcomes for care home residents.
